# Socioeconomic inequalities in the prevalence of chronic diseases and preventive care among adults aged 45 and older in Shaanxi Province, China

**DOI:** 10.1186/s12889-019-7835-5

**Published:** 2019-11-06

**Authors:** Sha Lai, Chi Shen, Xiaowei Yang, Xiaolong Zhang, Yongjian Xu, Qian Li, Jianmin Gao, Zhongliang Zhou

**Affiliations:** 1 School of Public Policy and Administration, Xi’an Jiaotong University, P.O Box 86, No. 76 West Yanta Road, Xi’an, 710061 Shaanxi China; 2 Jinhe Center for Economic Research, Xi’an Jiaotong University, No. 28 Xianning West Road, Xi’an, 710049 Shaanxi China; 3 the First Affiliated Hospital of Xi’an Jiaotong University, No.277 West Yanta Road, Xi’an, 710061 Shaanxi China

**Keywords:** Chronic disease, Hypertension, Diabetes, Preventive care, Inequality, China

## Abstract

**Background:**

Monitoring inequalities in chronic disease prevalence and their preventive care can help build effective strategies to improve health equality. Using hypertension and diabetes as a model, this study measures and decomposes socioeconomic inequalities in their prevalence and preventive care among Chinese adults aged 45 years and older in Shaanxi Province, an underdeveloped western region of China.

**Methods:**

Data of 27,728 respondents aged 45 years and older who participated in the fifth National Health Services Survey conducted in 2013 in Shaanxi Province were analyzed. The relative indexes of inequalities based on Poisson regressions were used to assess disparities in the prevalence of hypertension and diabetes and their preventive care between those with the lowest and the highest socioeconomic status, and the concentration index was used to measure the magnitude of the socioeconomic-related inequality across the entire socioeconomic spectrum. The contribution of each factor to the inequality was further estimated via the concentration index decomposition.

**Results:**

Our results indicate a higher prevalence of hypertension and diabetes among the rich than the poor individuals aged 45 years and older in Shaanxi Province, China. Among individuals with hypertension or diabetes, significant inequalities favoring the rich were observed in the use of preventive care, i.e. in adequate use of medication and of blood pressure/blood glucose monitoring. Furthermore, economic status, educational level, employment status, and urban-rural areas were identified as the key socioeconomic indicators for monitoring the inequalities in the patient preventive care.

**Conclusions:**

Our study suggests that the existence of clear inequities in the prevalence of chronic diseases and preventive care among adults aged 45 and older in Shaanxi Province, China. These inequalities in chronic diseases could be as much a cause as a consequence of socioeconomic inequalities.

## Background

Inequality in health refers to the differences in health conditions, or access to health care between groups, such that one group is better off than another. Differences in health between socioeconomic groups are one of the major public health challenges worldwide [1]. In China, for example, although average life expectancy is dynamically growing, inequalities in health still exist within the country due to ever increasing income disparity [2, 3]. Therefore, measuring inequalities in health, especially the gap between economic groups, is important for health scientists and policy-makers to improve health equality [4].

Studies conducted in high-income countries have indicated that socioeconomic inequality in the prevalence of chronic non-communicable diseases (NCDs) is the leading cause of inequalities in life expectancy and total mortality among the rich and the poor [5–7]. The increased emergence of NCDs also has serious economic and public health consequences, especially in many low- and middle-income countries [8]. In China, it is estimated that 80% of deaths and 70% of the disease burden are attributed to NCDs [9]. The negative effects of aging, environmental deterioration, unhealthy lifestyles and diets, and China’s rapid industrialization and urbanization create considerable challenges for China’s public health [10]. NCDs will become another significant public health threat if the trend continues.

Previous research has shown that socioeconomic status is the main determinant of chronic disease distribution in populations [11, 12]. However, the association between the socioeconomic status and the chronic disease varies widely among regions and even between different periods in the same region. For instance, the distribution of cardiovascular diseases across the socioeconomic status spectrum has reversed over time from a higher prevalence among individuals with a high socioeconomic status to a greater prevalence among individuals of low socioeconomic status [13, 14]. However, previous studies from China have shown a positive relationship between the prevalence of hypertension and diabetes and socioeconomic status [15, 16]. A comparative work on socioeconomic and educational gradient of the prevalence of several common NCDs across eight European countries has found that most NCDs have a higher prevalence among the lower educated, but the relationship between the prevalence of NCDs and the economic status is highly variable for different types of NCDs and countries [17]. The pattern of inequality in NCD prevalence in different countries or regions depends on the stage of their socioeconomic development and their health policies [18].

Moreover, disparities exist not only in NCDs prevalence, but also in self-care, medication adherence, and preventive care which affect the outcome of NCDs [19–21]. For instance, diabetic patients need to perform a complex set of preventive care tasks (e.g. self-monitoring blood glucose, using hypoglycemic drugs, changing eating habits) essential for controlling blood glucose and preventing complications and advanced-stage disease [22]. Despite the relatively low cost of these preventive care tasks, in most countries, individuals from the economically disadvantaged groups and communities are more likely to die due to chronic diseases than their richer counterparts [18].

Chinese government has recognized these challenges and responded to them. The new healthcare system reform, which was launched in 2009, is seen as a key step in efforts to address health inequalities in China [23]. The government’s plan was to reinforce the health care system, specifically to support the primary health care and promote equity in diagnoses and access to treatment [23]. Consequently, the basic public health package was designed to improve disease prevention and health care for vulnerable populations (e.g. elderly, women, children, and individuals with chronic health conditions) [24]. In 2009, the government provided 15 Yuan per head (raised to 35 Yuan in 2013, and 50 Yuan in 2017, 1 US$ = 6.5 Yuan) to healthcare providers to deliver basic public health services [25]. This package mainly included establishment of health records, health education/promotion, geriatric care, chronic disease management. Effective measures have been applied to the prevention, management and control of NCDs. However, whether preventive care measures associated with chronic diseases were widely used and equitable has not been sufficiently investigated, especially in the underdeveloped western region of China.

This study aims to examine the socioeconomic-related inequalities in the prevalence of NCDs and in the use of preventive care among middle aged and older adults in Shaanxi Province of China and to quantify the contribution of different determinants to the inequality.

## Methods

### Data sources

We used a dataset from a cross-sectional household health survey conducted in Shaanxi Province in 2013. This survey was a part of the fifth National Health Services Survey (NHSS) and was the largest health survey in Shaanxi Province, an underdeveloped region in western China. Shaanxi Province covers an area of about 205,800 km^2^ and has a population of 37.6 million in 2013, among which people aged 45 years and older account for about 33.5% [26].

A structured questionnaire was used to conduct face-to-face interviews with all members of selected households. Questionnaires collected data about demographic characteristics, the economic status (annual household expenditure), other socioeconomic information (e.g. educational level, employment status), and health status, including information about chronic conditions and preventive care specific to hypertension and diabetes. In our survey, respondents were required to answer questions on their own and proxy responses by familiar family members were only used when the respondents were unable to express themselves accurately.

A four-stage stratified cluster random sampling approach was used to select the representative survey respondents in Shaanxi Province. In short, 32 districts or counties were stratified, among which 160 sub-districts or townships were randomly selected. Next, 320 communities or villages were randomly selected from these sub-districts or townships. Finally, 20,700 households were randomly selected, and every family member of these households (in accordance with census register information) was interviewed, adding to a total of 57,529 surveyed individuals. In the process of data collection, extensive quality control measures were conducted as previously described [27]. In our study, we only analyzed data from a total of 27,728 individuals aged 45 years and older, focusing on an age group more likely to be affected by chronic diseases.

### Variables

The variables used in this paper included: a) the presence of hypertension/diabetes and the behavior of preventive care for those with hypertension/diabetes (these are the outcome variables), b) economic status, c) demographic and other socioeconomic factors.

- the presence of hypertension/diabetes and the preventive care behavior

Hypertension and diabetes were selected as model examples of chronic disease in this study because they represent the two most common diseases and the main causes of death and disease burden in China [9, 10]. Furthermore, adequate use of medication and monitoring blood pressure/blood glucose were the two indicators selected as proxies of patient preventive care behavior because these two indicators are the main means of preventing and monitoring complications and advancement of both diseases.

The outcome variables in the study were all binary. The first set of variables was the presence of hypertension and/or diabetes. The presence of hypertension/diabetes was determined from self-reports of physician-diagnosed hypertension/diabetes from answers to questions such as “Have you ever been diagnosed with hypertension?” and “Have you ever been diagnosed with diabetes?” Next, if the respondents had hypertension/diabetes, they were asked whether they made an adequate use of medication according to their physician’s instructions and whether their blood pressure/blood glucose had been monitored in the last three months.

(b) the economic status

The household consumption expenditure per equivalent adult was used as a proxy measure of economic status in this study as proposed by previous studies [28]. It was derived by dividing household consumption expenditure by the equivalent number of adults in the household which has been described in detail before [29, 30]. For the regression analysis, the economic status variable was divided into quintiles according to the household consumption expenditure per equivalent adult as follows: poorest (i.e. the lowest 20%, ≤ 5030 Yuan), poorer (lower 20%, 5031–7704 Yuan), middle (middle 20%, 7705–10,976 Yuan), richer (higher 20%, 10,977–16,651 Yuan) and richest (highest 20%, ≥ 16,652 Yuan).

(c) the demographic and other socioeconomic variables

The demographic characteristics included were gender and age. Age was categorized into three groups: 45–64, 65–79, and 80 or above. Other socioeconomic characteristics included were educational level, employment status, marital status, basic medical insurance (no or yes), commercial medical insurance (no or yes), and urban or rural areas. Educational level was categorized into four groups: primary or below education, middle school, high school, and college or above education. Two employment status categories were employment and unemployment. Two marital status categories were married and single (including unmarried, divorced, separated and widowed). The basic medical insurance refers to China’s public insurance programs including Urban Employee Basic Medical Insurance, New Rural Co-operative Medical Scheme and Urban Residents Basic Medical Insurance. These three insurance programs cover more than 98% of the population in China.

### Statistical analysis

The data analyses performed in the study included a descriptive analysis, a simple weighted point estimate, an estimation of the relative index of inequality (RII), a calculation of the economic-related concentration index (C), and a further decomposition analysis for the C. The RII and C are widely used to estimate a magnitude of inequality in health or healthcare [31, 32]. RII compares extremes and C summarizes inequality across the entire socioeconomic spectrum. All analyses were performed independently for hypertension and diabetes.

- the relative index of inequality

The RII, mainly used in public health and epidemiology, is a regression-based index of inequality used to compare rates of disease prevalence between those with the lowest and the highest socioeconomic status [31]. RII can be interpreted as the ratio of the estimated prevalence of disease between the poorest and the richest. Therefore, a RII value greater than one signifies a higher prevalence among those with the lowest socioeconomic status and vice versa. Given that binary outcomes, Poisson regressions with robust error variance were used to generate the prevalence rate ratio estimates across the economic groups adjusted for confounding variables (i.e., RIIs) in a cross-sectional study, as suggested in previous reports [33]. RIIs were reported in two stages: first RIIs were adjusted for age and gender, while second RIIs were additionally adjusted for educational level, occupational status, and other socioeconomic factors.

(b) the concentration index

The C captures the socioeconomic-related inequalities in health or health care and gives a measure of the magnitude of inequality across the entire socioeconomic spectrum [32]. The C ranges from − 1 to 1, with an index of 0 equivalent to perfect equality. A positive C signifies that a health or health care variable is more concentrated among the richer population and vice versa. The C formula is as follows:

1 $$ \mathrm{C}=\frac{2}{\mu}\mathit{\operatorname{cov}}\left({y}_i,{r}_i\right) $$

Where y is the health or health care variable (e.g. hypertension/diabetes prevalence and preventive care in this study), μ is the mean of the health or health care variable, *r*_*i*_ is the fractional rank of the *i* th individual in the economic distribution, ranging from 0 to 1.

(c) the decomposition analysis for C

Wagstaff et al. proved that the C can be decomposed into its contributing demographic and socioeconomic factors, where the contribution of each factor is the product of the degree of economic-related inequality in that factor and the sensitivity of the health or health care outcome variable with respect to that factor [34].

Since the outcome variables analyzed in our study were binary variables, probit regressions were used to calculate the partial effects of each explanatory variable and the results should not be used to infer a direction of causality [35]. All explanatory variables in regressions were categorical dummy variables. Health (or a health care outcome variable) (y) is modelled as follows:

2 $$ {y}_i={\sum}_k{\beta}_k{x}_{ki}+{\varepsilon}_i $$

where *β*_*k*_ are the partial effects, dy/dx of each regressor is evaluated at the sample mean; and ε is the error term. *x*_*k*_ are a set of explanatory variables.

The concentration index C(y) can be decomposed as:

3 $$ \mathrm{C}={\sum}_k\left(\frac{\beta_k{\overline{x}}_k}{\mu}\right){C}_k+\frac{GC_{\varepsilon }}{\mu } $$

where *β*_*k*_ are the partial effects of the k regressors (i.e. explanatory variables), taken from Eq. (2). ${\overline{x}}_k$ are the means of each regressor and μ is the mean of the health or health care variable. *C*_*k*_ is the concentration index of each regressor and *GC*_*ε*_ is the generalized concentration index of ε. The residual component ($\frac{GC_{\varepsilon }}{\mu }$) represents the inequality that is not explained by the regressors. The deterministic component (${\sum}_k\left(\frac{\beta_k{\overline{x}}_k}{\mu}\right){C}_k$) focuses on two elements. These are the degree of unequal distribution of each regressor across the economic spectrum (*C*_*k*_) and the elasticity of health with respect to the regressor$, \kern0.5em \left({\eta}_k={\beta}_k\frac{{\overline{x}}_k}{\mu}\right)$. We calculated the absolute contribution of each regressor (*Q*_*k*_ = *η*_*k*_*C*_*k*_). The contribution of each regressor can take both positive and negative values. According to Eq. (3), even if an explanatory variable has a massive effect on the health or health care variable, if the variable is equally distributed between the rich and the poor, then the explanatory variable is not a key source of inequality. Then we calculated the percentage contribution of each regressor (100*Q*_*k*_ /C). Of note, the negative and positive contributions may cancel out in the aggregate and the percentage contribution of the regressors and error term sum would be 100%, so the percentage contribution of several regressors may represent large positive and negative contributions, even over 100%.

Inequalities in health or health care are associated with demographic factors, e.g. age, and socioeconomic-related factors, e.g. economic resources and urban-rural indicators. Policy makers may be more focused on inequalities arising from socioeconomic-related factors, because some of the demographic factors are inevitable [35]. In this study, age-sex adjusted C was calculated by subtracting the contributions of age and gender from the total C based on the decomposition results [36, 37].

Sampling weights were used to account for the sampling design and to ensure the results represented the population of Shaanxi Province as previously described [38]. Svyset command in STATA version 14.0 was used to specify the design for all analytical models. Furthermore, we adjusted standard errors for clustering at the family level for all models.

## Results

### Distribution of variables

Our study included a total of 27,728 respondents aged 45 and older. The overall response rate was above 85% including proxy interviews. Excluding proxy interviews, the response rate was above 75%. 5% of the households were re-interviewed with eight same questions to check for consistency, and the consistency between survey and re-interviewed survey was over 95%. Myer’s Blended Index was used to measure the quality of self-reported age data [39]. There were no obvious digit preference nor age heaping in the ages data (Myer’s Blended index = 1.62). Table 1 represents the descriptive statistics of each variable used in this paper. The prevalence of hypertension and diabetes was 21.0 and 4.1%, respectively. Among the individuals with hypertension, 61.1% of the patients took adequate medication and 92.7% of the patients had their blood pressure measured in the three months preceding the survey. Among the respondents with diabetes, 74.2% of the patients took adequate medication and 86.3% of the patients had their blood glucose tested in the three months prior to the survey.

**Table 1:** Characteristics of respondents aged 45 years and older (*n* = 27,728)

Variables	*N*	Proportion	SE
NCDs and preventive care			
Hypertension and preventive care			
Presence of Hypertension	5742	0.210	0.003
Adequate medication	3360	0.611^a^	0.007
Measure blood-pressure	5303	0.927^a^	0.004
Diabetes and preventive care			
Presence of Diabetes	1055	0.041	0.001
Adequate medication	765	0.742^b^	0.014
Test blood-glucose	906	0.863^b^	0.011
Demographic Variables			
Age Group			
45–64	19,809	0.707	0.003
65–79	6842	0.253	0.003
80 or above	1077	0.040	0.001
Gender			
Female	14,018	0.506	0.003
Male	13,710	0.494	0.003
Economic Status			
Poorest	5547	0.191	0.002
Poorer	5548	0.196	0.002
Middle	5591	0.199	0.003
Richer	5560	0.205	0.003
Richest	5482	0.208	0.003
Education Level			
Primary or below	14,838	0.499	0.003
Middle school	8709	0.326	0.003
High School	3428	0.142	0.002
College or above	753	0.033	0.001
Employment Status			
Unemployed	8559	0.328	0.003
Employed	19,169	0.672	0.003
Marital Status			
Single	4175	0.145	0.002
Married	23,553	0.855	0.002
Basic Medical Insurance			
No	267	0.012	0.001
Yes	27,461	0.988	0.001
Commercial Medical Insurance			
No	26,183	0.942	0.002
Yes	1545	0.058	0.002
Urban/Rural			
Rural	17,922	0.606	0.003
Urban	9806	0.394	0.003

Note: (1) Numbers were unweighted, proportions were weighted; (2) ^a^ The proportions of preventive care among people with hypertension; (3) ^b^ The proportions of preventive care among people with diabetes

### Poor-rich distribution of NCDs and their preventive care

Table 2 reports the prevalence of hypertension and diabetes and the proportion of preventive care among people living with hypertension or diabetes ranked into economic quintiles, the RII and the economic-related C. The RII reflected the disparities between the lowest and the highest economic groups and the socioeconomic-related C reflected the inequalities across the entire economic spectrum.

**Table 2:** Proportion of chronic diseases and preventive care (%) by economic quintile, and economic-related inequality

	Hypertension and preventive care			Diabetes and preventive care		
	Presence of Hypertension	Adequate medication	Measure blood-pressure	Presence of Diabetes	Adequate medication	Test blood-glucose
Respondents	27,728	5742	5742	27,728	1055	1055
Poorest, % (95%CI) ^a^	18.18 (17.13–19.23)	50.99 (47.99–54.00)	89.30 (87.44–91.16)	1.69 (1.34–2.05)	59.09 (52.16–66.01)	77.52 (71.64–83.4)
Poorer, % (95%CI) ^a^	20.06 (18.97–21.16)	56.89 (53.92–59.86)	91.05 (89.3–92.81)	2.97 (2.48–3.45)	68.77 (62.30–75.24)	82.73 (77.41–88.05)
Middle, % (95%CI) ^a^	20.87 (19.74–22.00)	58.94 (55.96–61.92)	90.36 (88.56–92.17)	3.75 (3.20–4.29)	78.58 (72.80–84.36)	87.00 (82.08–91.91)
Richer, % (95%CI) ^a^	21.43 (20.28–22.58)	64.50 (61.62–67.37)	94.86 (93.56–96.17)	4.40 (3.81–4.99)	80.07 (74.46–85.68)	91.36 (87.39–95.33)
Richest, % (95%CI) ^a^	24.18 (22.96–25.41)	72.10 (69.41–74.78)	97.03 (96.1–97.95)	7.44 (6.68–8.21)	82.81 (77.32–88.3)	91.59 (87.59–95.60)
RII^b^, (95% CI)	0.70*** (0.64–0.75)	0.70*** (0.65–0.75)	0.92*** (0.90–0.94)	0.21*** (0.16–0.27)	0.72*** (0.63–0.83)	0.85*** (0.78–0.93)
RII^c^, (95% CI)	0.73*** (0.67–0.79)	0.81*** (0.75–0.87)	0.93*** (0.90–0.95)	0.32*** (0.25–0.41)	0.80*** (0.69–0.92)	0.87*** (0.80–0.96)
C, (95%CI)	0.056*** (0.041–0.070)	0.068*** (0.054–0.081)	0.017*** (0.013–0.022)	0.264*** (0.228–0.299)	0.065*** (0.043–0.088)	0.036*** (0.021–0.051)

Note: (1) 95% CI: 95% confidence interval, C: concentration index, RII: relative index of inequality; (2) ^a^: weighted but unadjusted prevalence estimates; (3) ^b^: RII values were established by Poisson regressions with robust variance and adjusted for gender and age; (4) ^c^: RII values were established by Poisson regressions with robust variance and adjusted for gender, age, education level, marital status, working status, basic medical insurance, commercial medical insurance and urban/rural area; (5) ***p < 0.001

According to the RIIs adjusted for age, gender, education level and other socioeconomic variables, the hypertension and diabetes prevalence ratios of the most deprived relative to the most advantaged were 0.73 (95% CI: 0.67–0.79) and 0.32 (95% CI: 0.25–0.41), respectively. The positive values of Cs (hypertension: C = 0.056; 95% CI, 0.041–0.070; diabetes: C = 0.264; 95% CI, 0.228–0.299) suggest that hypertension and diabetes are concentrated toward the economically advantaged groups.

Among respondents with hypertension or diabetes, the adjusted prevalence ratio of the poorest relative to the richest was 0.81 (95% CI: 0.75–0.87)/0.80 (95% CI: 0.69–0.92) for taking adequate medication to control high blood pressure/blood glucose and 0.93 (95% CI: 0.90–0.95)/0.87 (95% CI: 0.80–0.96) for monitoring blood pressure/blood glucose. The positive values of Cs indicate the existence of inequalities favoring the rich in preventive care for hypertension and diabetes patients.

### Decomposition of the Cs

Table 3 reports the detailed decomposition of Cs for hypertension and diabetes prevalence and patient preventive care. The partial effect estimates, and the absolute contribution and percentage contribution of each determinant are presented.

**Table 3:** Decomposition of concentration index of chronic diseases and preventive care

	Hypertension and preventive care									Diabetes and preventive care								
	Presence of hypertension			Adequate medication			Measure blood-pressure			Presence of diabetes			Adequate medication			Test blood-glucose		
	dy/dx	Con.	% con.	dy/dx	Con.	% con.	dy/dx	Con.	% con.	dy/dx	Con.	% con.	dy/dx	Con.	% con.	dy/dx	Con.	% con.
Age (Ref: 45–64 years)																		
65–79	0.137***	−0.012	−21.321	0.077***	− 0.002	−2.787	0.025***	< 0.001	− 2.264	0.018***	− 0.008	−3.161	0.007	< 0.001	− 0.056	0.021	< 0.001	− 0.254
80 or above	0.129***	−0.002	−3.539	0.055*	< 0.001	−0.469	−0.011	< 0.001	0.233	< 0.001	< 0.001	−0.005	0.091	< 0.001	0.311	−0.072	< 0.001	−0.379
Gender (Ref: Female)																		
Male	−0.043***	0.001	1.023	−0.051***	−0.001	−1.587	− 0.006	< 0.001	− 0.473	−0.001	< 0.001	0.026	0.017	0.001	0.987	0.007	< 0.001	0.644
Economic status (Ref: Poorest)																		
Poorer	0.039***	−0.015	−27.020	0.052**	−0.007	−10.499	0.012	−0.001	−6.139	0.020***	−0.042	−15.964	0.050	−0.006	−8.413	0.022	−0.002	−5.823
Middle	0.046***	< 0.001	0.154	0.052**	< 0.001	0.014	0.005	< 0.001	0.003	0.026***	< 0.001	0.101	0.132***	< 0.001	< 0.001	0.059**	< 0.001	< 0.001
Richer	0.046***	0.018	32.382	0.083***	0.011	16.805	0.039***	0.003	19.252	0.028***	0.060	22.873	0.118***	0.013	19.872	0.082***	0.008	21.208
Richest	0.071***	0.054	97.799	0.132***	0.036	53.332	0.057***	0.010	56.894	0.052***	0.217	82.167	0.134***	0.030	45.182	0.088***	0.016	45.756
Educational level (Ref: Primary or below)																		
Middle school	−0.034***	−0.004	−6.498	0.038**	0.001	2.173	0.006	< 0.001	0.894	0.001	< 0.001	0.158	0.046	0.001	0.973	0.005	< 0.001	0.172
High school	−0.021**	− 0.004	−6.548	0.056**	0.004	5.527	−0.004	< 0.001	−1.013	0.003	0.003	1.004	0.019	0.001	1.905	0.033	0.002	5.051
College or above	0.007	0.001	1.146	0.090**	0.003	4.455	0.056***	0.001	6.760	0.007	0.003	1.201	0.028	0.001	2.158	0.056	0.002	6.733
Employment status (Ref: Unemployed)																		
Employed	−0.079***	0.007	11.745	−0.096***	0.005	7.342	−0.003	< 0.001	0.648	−0.033***	0.015	5.559	−0.109***	0.005	7.025	−0.104***	0.004	10.311
Marital status (Ref: Single)																		
Married	−0.005	< 0.001	−0.766	0.072***	0.002	3.527	< 0.001	< 0.001	−0.016	0.005	0.002	0.853	0.085**	0.002	2.332	0.027	< 0.001	1.132
Basic medical insurance (Ref: No)																		
Yse	0.007	< 0.001	−0.126	−0.082	0.001	0.819	0.031	< 0.001	−0.774	−0.003	< 0.001	0.052	0.033	< 0.001	−0.226	0.050	< 0.001	−0.525
Commercial insurance (Ref: No)																		
Yes	0.033***	0.001	2.238	−0.060*	−0.001	− 0.913	0.014	< 0.001	0.521	−0.004	− 0.001	− 0.342	−0.071	< 0.001	< 0.001	0.016	< 0.001	< 0.001
Urban and rural (Ref: Rural)																		
Urban	0.004	0.001	2.282	0.096***	0.014	20.265	0.012	0.001	6.311	0.016***	0.030	11.477	0.099***	0.013	19.794	−0.017	−0.002	−5.296

Note: (1) dy/dx: Partial effect in Probit regression model; Con.: The absolute contribution of each determinant (${Q}_k={\beta}_k\frac{{\overline{x}}_k}{\mu }{C}_k$); % con.:The percentage contribution of each determinant to the total concentration index (100*Q*_*k*_ /C); (2) *p < 0.05, **p < 0.01, ***p < 0.001

The partial effects (dy/dx) of each explanatory variable on the presence of hypertension/diabetes and the preventive care behavior were estimated by running probit regressions based on Eq. 2. As can be seen from the partial effect estimates, the economic status was significant and positively associated with the presence of hypertension and diabetes (e.g. the partial effect estimates of poorer, middle, richer, and richest on the presence of hypertension were 0.039, 0.046, 0.046 and 0.071, respectively, and all were significant); among those with hypertension or diabetes, the economic status was also significant and positively associated with preventive care, i.e. with adequate use of medication and monitoring of blood pressure/blood glucose. Specifically, the respondents in the higher economic status groups were more likely to suffer from hypertension or diabetes, and respondents with hypertension or diabetes in the higher economic status groups were more apt to have better preventive care behavior. The education level was found to be significant in hypertension prevalence and preventive care, but with mixed signs on partial effects. Employment was found to be significant and negatively associated with hypertension or diabetes prevalence and their preventive care. The respondents with hypertension or diabetes who lived in urban areas were more likely to use medication adequately. The basic medical insurance status, a variable particularly interesting for policy making, was not found to be statistically significantly associated with hypertension and diabetes prevalence and preventive care.

The absolute and percentage contributions to the total economic-related inequality of each explanatory variable were calculated based on Eq. 3. The absolute contribution depends both on the impact of each determinant on health or health care and on the degree of the unequal distribution across the economic spectrum. A positive (negative) absolute contribution of each determinant reveals that the total inequality would be lower (higher) if that determinant had no impact on health, or was evenly distributed across the economic gradient.

We illustrated and explained the mechanics of the decomposition using an ‘employed’ variable on the hypertension prevalence. The absolute contribution to the total C of the ‘employed’ variable was 0.007, calculated by multiplying its partial effect by its mean and C, then dividing by the mean of hypertension prevalence (i.e. ${Q}_k={\beta}_k\frac{{\overline{x}}_k}{\mu }{C}_k$). Its percentage contribution (11.745%) equals its absolute contribution (0.007) divided by the concentration index of the hypertension prevalence (0.056 in Table 2) (i.e. 100*Q*_*k*_ /C). The policy implications of these results are that economic-related inequality in hypertension prevalence could be improved by reducing disparity in the distribution of employment across the economic spectrum, e.g. by increasing employment opportunities among the economically disadvantaged groups; policies for hypertension prevention should be provided for unemployed individuals to improve their health.

Concluding from the contributions of each demographic and socioeconomic characteristic to the inequalities in the prevalence of hypertension or diabetes it can be said that the three largest contributors to the inequalities were the economic status, employment, and urban-rural area. Among individuals with hypertension or diabetes, economic status, urban-rural area, educational level, and employment played major contributory roles to inequalities favoring the rich in the preventive care, i.e. in adequate use of medication and monitoring of blood pressure/blood glucose. The economic status was revealed to have the largest contribution to both the inequalities in the prevalence of hypertension or diabetes and the inequalities favoring the rich in their preventive care. The contribution of the economic status depended both on its large impact on the prevalence of both diseases and their preventive care and on its unequal distribution.

Table 4 summarizes the contributions to Cs and age-sex adjusted Cs in the prevalence of hypertension and diabetes and the patient preventive care. The contribution of the age-sex group was calculated by aggregating the contribution of age and sex dummy variables using the same calculations as the other groups, i.e., economic status and other factors. Table 4 shows that the largest contributor was the economic status (57–103%) and that contributions of the other sociological factors were of a lower importance (3–43%). Age and gender played less important roles in contributing to the observed total inequalities. The age-sex adjusted Cs in the prevalence of hypertension and diabetes were 0.069 and 0.272, respectively. In other words, even when controlled for gender and age differences, there was a higher prevalence of hypertension and diabetes among the rich compared to the poor. The age-sex adjusted Cs for adequate use of medication to control blood pressure/blood glucose were 0.071/0.064; and the age-sex adjusted Cs for monitoring of blood pressure/blood glucose were 0.017/0.036. That is, even when controlled for gender and age differences, there were clear inequalities favoring the rich in the preventive care for individuals with hypertension or diabetes.

**Table 4:** Summary of contributions of factors and age-sex adjusted Cs

	Hypertension and preventive care			Diabetes and preventive care		
	Presence of Hypertension	Adequate medication	Measure blood-pressure	Presence of Diabetes	Adequate medication	Test blood-glucose
Age-sex groups, Con. (% con.)	−0.013 (−23.837%)	− 0.003 (−4.843%)	< 0.001 (−2.504%)	−0.008 (−3.140%)	0.001 (1.242%)	< 0.001 (0.011%)
Economic status, Con. (% con.)	0.057 (103.315%)	0.040 (59.652%)	0.012 (70.010%)	0.235 (89.177%)	0.037 (56.641%)	0.022 (61.141%)
Other factors, Con. (% con.)	0.002 (3.473%)	0.029 (43.195%)	0.002 (13.331%)	0.052 (19.962%)	0.023 (33.961%)	0.006 (17.578%)
Residual, Con. (% con.)	0.010 (17.049%)	0.002 (1.996%)	0.003 (19.163%)	−0.015 (−5.999%)	0.004 (8.156%)	0.008 (21.270%)
C	0.056	0.068	0.017	0.264	0.065	0.036
Age-sex adjusted C	0.069	0.071	0.017	0.272	0.064	0.036

Note: Con.: The absolute contribution of determinants to concentration index; % con.:The percentage contribution of determinants to the total concentration index; C: concentration index

## Discussion

This study is the first to explore the socioeconomic-related inequalities in the prevalence of NCDs and the patient preventive care in Shaanxi Province, China, and further quantifies the contribution of several selected determinants toward these inequalities. Our results suggest a higher prevalence of hypertension and diabetes among the rich compared to the poor, and clear inequalities in the preventive care favoring the rich (i.e. adequate use of medication and monitoring of blood pressure/blood glucose) among individuals with hypertension or diabetes in Shaanxi Province, China. A decomposition analysis revealed that the inequalities in the prevalence of hypertension and diabetes and patient preventive care were mostly driven by differences in economic status and, additionally, by other socioeconomic factors (i.e. educational level, employment status, and urban or rural areas) and unobserved effects.

Our results reveal that the prevalence of hypertension and diabetes are concentrated toward economically advantaged groups. These findings are consistent with previously published literature on other regions of China based on self-reported physician-diagnosed data [16] or anthropometric data (i.e. an oral glucose tolerance test and measurement of blood pressure) [15, 40, 41]. Findings from some low- and middle-income countries also show consistent economic gradients [42–44]. However, some studies, mostly from high-income countries, indicate an inverse economic gradient [45–47]. Furthermore, a few studies have revealed that the association between socioeconomic status and the prevalence of hypertension or diabetes can change or even reverse over time, especially in low- and middle-income countries [13, 14]. In China, which is now in a period of rapid economic growth and globalization, higher socioeconomic groups appeared to be at a higher risk of hypertension and diabetes, partly due to a westernized lifestyle leading to an unhealthy diet, physical inactivity, and obesity [48]. The pattern of inequality in the prevalence of NCDs depends on the level of development of social, economic, and health policies [18]. As such, this study provides a glimpse into the underdeveloped western areas of China, given the socioeconomic disparities in the prevalence of hypertension and diabetes.

For patients with hypertension or diabetes, strengthening the preventive care implies reducing complications and significantly improving their chances of survival and their well-being. However, our results indicate that, among individuals with hypertension or diabetes, clear inequalities exist in the preventive care favoring the rich. These results possibly support some previous findings that NCDs patients of low socioeconomic status are more apt to get worse outcomes [18, 49]. Our results also identify several key socioeconomic variables associated with the preventive care for individuals with hypertension or diabetes. Economic status, level of education, employment status, and urban-rural areas are the key socioeconomic indicators for monitoring inequalities in the patient preventive care as demonstrated by previous studies, such as Yusuf et al. [19], Carrieri et al. [50], and Gopichandran et al. [51]. A variety of social and cultural factors associated with an individual’s socioeconomic status have an impact on one’s health beliefs; in turn, these beliefs can be vital for determining the use of preventive care [52].

Our study has not identified a significant correlation between basic medical insurance and the preventive care for patients with hypertension or diabetes, even though studies from other countries have suggested that implementation of universal health insurance could be an effective way to improve treatment rates among those with chronic conditions and to reduce socioeconomic gradients [18, 20]. In China, because of limited financing, the basic medical insurance is primarily oriented toward inpatient care, outpatient care for catastrophic diseases, and chronic disease-created complications. As a result, the effectiveness of the basic medical insurance in increasing preventive care for patients with NCDs could be limited. Encouragingly, the basic health insurance has advanced toward the tendency of extending its coverage to general outpatient care and more types of NCDs, which may reduce the inequality in patient preventive care to some extent. The effect of China’s basic medical insurance on the use of preventive care among NCDs patients will require further extensive studies.

The basic public health package launched in 2009 is quite likely to help decrease inequalities in diagnosis, preventive care, and outcome for patients with NCDs in China. Previously published studies from other countries have shown that universally accessible primary care helps to achieve glucose control and reduce complications in diabetics [53]. In recent years, Chinese government has provided continuous financial support for primary healthcare institutions to offer public health services. For instance, for elderly people, the government provides a free physical exam every year, guidance on self-care/self-help, and injury prevention; for hypertension patients, the government provides at least one free follow-up visit every three months which includes health evaluation, syndrome surveillance, behavioral intervention, guidance on the use of medicines, and health education; similarly, for diabetes patients, the government provides free blood glucose tests every three months and other visiting services [25].

Strong policies and programs should be considered to address inequalities in the prevalence of NCDs and in secondary prevention in patients already suffering from NCDs. Our findings suggest that economic status is the main source of inequalities in the prevalence of NCDs and in secondary prevention. For patients living with chronic conditions, economic obstacles may deter their use of preventive care and further affect the outcome of the chronic disease; in turn, increasing medical expenditures can worsen a family’s economic situation, reducing them to poverty.

Although China has made considerable progress in universal healthcare and primary healthcare, several challenges remain. Firstly, the basic medical insurance system should further expand the scope of reimbursement to remove economic barriers to access health care by enrollees with chronic conditions and to reduce the economic burden on NCDs patients. Powerful policies have been adopted in the public health package, but it is estimated that the basic public health package covered only a half of the elderly population aged over 65 years (57.1 million) and about one-fifth of the patients with hypertension and diabetes (35.5 million patients with hypertension and 9.2 million patients with diabetes) in 2010 [23]. Hence, secondly, primary health care and public health services should be strengthened and cover more of the target population, especially the disadvantaged communities, to improve early detection and treatment through public education and interventions. For example, Farzadfar et al. have introduced a successful program in Iran’s primary health care system for controlling and managing hypertension and diabetes [53]. Thirdly, it is equally essential to improve the physical accessibility of primary care and enhance the quality of health services, especially in rural areas. Implementing high quality and equitable primary care and ensuring availability and low cost of medicines essential for prevention and early treatment of NCDs are examples of effective ways to reduce NCDs burdens and inequalities [18].

This study has several limitations that must be acknowledged. Firstly, the prevalence of hypertension and diabetes may be underestimated due to the self-reported physician-diagnosed format, which could affect the magnitude of inequality if the misdiagnosis mostly exists in the poor. However, hypertension and diabetes are less likely to be underestimated compared to other chronic diseases, because they are the focus of public health policy in China. That is, the government has offered continuous financial support for the primary health care for urban and rural residents to diagnose and prevent hypertension and diabetes. Moreover, our results on the relationship between the economic status and the prevalence of self-reported hypertension or diabetes are consistent with other findings based on anthropometric data for individuals in other regions of China [15, 40, 41, 48]. Despite the limitations of self-reported diagnoses, this study can build an evidence base for understanding the socioeconomic-related inequalities in the prevalence of hypertension and diabetes. Secondly, in order to obtain a representative sample at a lower cost, a complex sampling technique was conducted to select the survey respondents. The complex sampling design complicates the estimates. Thirdly, for binary health variables, the use of C captures the magnitude of the socioeconomic-related inequalities across the entire socioeconomic spectrum but without considering the upper bound of the variable. Fourthly, recall biases are inevitable in questionnaire-based surveys, especially those pertaining to behaviors several months prior to the survey. Nevertheless, these large survey data sets enable us to identify socioeconomic-related inequalities in preventive care for patients in the underdeveloped western areas of China. Future studies should focus on monitoring the changes in the inequalities in the prevalence of NCDs and their preventive care and on assessing the effectiveness of health policies in mitigating these inequalities.

## Conclusions

Monitoring inequalities in the prevalence of NCDs and patient preventive care can help design effective strategies to improve health equality. Our results indicate a greater prevalence of hypertension and diabetes among the rich than the poor and clear inequalities in the preventive care favoring the rich (adequate use of medication and monitoring blood pressure/blood glucose) among individuals with hypertension or diabetes. Importantly, the inequalities in the prevalence of NCDs and their preventive care could be as much a cause as a consequence of the socioeconomic-related inequalities. Economic status, educational level, employment status, and urban-rural areas are the key identified socioeconomic indicators for monitoring the inequalities in the patient preventive care in Shaanxi Province, China.

## Data Availability

The data used in this study belong to the Shaanxi Health Commission (SHC) and contain the personal information (e.g., name, ID, etc.) of participants. The authors were involved in data collection. Due to the sensitive nature of these data and restrictions imposed by the SHC, the authors cannot make these data publicly available. Other researchers who want to use the data may submit requests for data access to the SHFPC at sxwjwwz@126.com.

## Abbreviations

95% CI: 95% confidence interval
C: Concentration index
NCD: Non-communicable disease
NHSS: National Health Services Survey
RII: relative index of inequality
